# The Hematological and Social Factors Affecting Length of Stay in Acute Stroke Patients: A Quality Assessment Study From a Single Institute

**DOI:** 10.7759/cureus.74411

**Published:** 2024-11-25

**Authors:** Aparna Suryadevara, Asad Khan, Vivian Hamlett, Vamshi Krishna Sai Balasetti, Tijil Agarwal, Ninh Doan

**Affiliations:** 1 Internal Medicine, Hospital Physician Services Southeast-PC South, Baptist Medical Center South, Montgomery, USA; 2 Vascular and Interventional Neurology, Baptist Health Neuroscience Partners Neurology, Baptist Medical Center South, Montgomery, USA; 3 Neurology, Baptist Health Neuroscience Partners Neurology, Baptist Medical Center South, Montgomery, USA; 4 Neurosurgery, Baptist Health Neuroscience Partners Neurosurgery, Baptist Medical Center South, Montgomery, USA

**Keywords:** anemia, factors, length of stay, obesity paradox, platelets, stroke

## Abstract

Introduction: Stroke is one of the common causes of mortality. The length of stay (LOS) for a stroke is a quality indicator and affects mortality. However, there are no large studies evaluating the LOS in an acute inpatient setting for stroke patients, mainly hematological and social parameters. In this quality assessment observational study, we analyzed the LOS in acute stroke patients at our institute.

Methods: We measured the LOS and divided the study population into two arms, arm A and arm B, to include patients with LOS≤10 and >10 days, respectively. We analyzed various factors affecting LOS and compared the two study arms and the literature.

Results: There were 483 patients with acute stroke in our study. The median LOS was 7.2±6.4 days (range 0.1 to 49.7). Patient age (p<0.00001), female gender (p<0.00001), hemorrhagic stroke (p=0.03), independence with ambulation before stroke (p<0.00001), type of placement (p<0.00001), type of insurance (p=0.0002), obesity (p 0.01) and anemia (p<0.00001), affected the LOS in our study. Most patients in arm B had medical complications (66%) during hospitalization.

Conclusions: In our quality assessment study, we observed that certain factors were associated with longer lengths of stay (LOS) among patients. These included geriatric age, female gender, hemorrhagic stroke, lack of ambulation independence, specific social determinants, discharge disposition to a facility, Medicare/Medicaid insurance, non-obesity, and hematological parameters such as anemia. The majority of these patients had additional medical complications. Thrombocytosis has been linked to reduced mortality in hemorrhagic stroke in the literature. However, our study found that platelet count does not significantly impact the length of stay (LOS) in ischemic stroke patients.

## Introduction

Stroke is the third most common cause of mortality in US (United States) population, excluding coronavirus disease (COVID) and accidents, as per Centers for Disease Control and Prevention (CDC) data [1]. The average length of stay for a stroke (LOS) patient is variable in literature but is about five to seven days and is a quality indicator in stroke patients [2,3]. Different factors affecting acute LOS in stroke patients in the literature include age, gender, the severity of stroke assessed by National Institutes of Health Stroke Scale (NIHSS) score, social factors like living alone, activities of daily living (ADL) dependent before the stroke, medical comorbidities like obesity, diabetes mellitus, heart disease, and dementia [4,5]. The LOS in inpatient rehab depends on ethnicity, which was not evaluated in acute stroke admissions [6]. The type of stroke, ischemic versus hemorrhagic, and involvement of anterior versus posterior circulation also affect the LOS [4,7]. Acute complications during hospitalization for acute stroke, like deep venous thrombosis/pulmonary embolism (DVT/PE), infections, falls, and constipation, also affect LOS and mortality at one month and twelve months post-stroke [8]. Admission and treatment for acute stroke on the medical floor versus the stroke unit also affect the LOS [9]. The LOS for acute stroke may vary depending on the type of health insurance, but it has not been evaluated in the past. It was evaluated in trauma patients in the past, showing Medicare/Medicaid patients having prolonged LOS than other insured patients, leading to an increased financial burden to institutes [10].

The LOS has a significant impact on stroke mortality, as prolonged hospital stays are often associated with increased complications and a higher risk of death in stroke patients [11]. Ethnicity was assessed in larger studies of stroke patients in other countries but not in the US [6,12]. Obesity in relation to stroke was assessed in only one study. Elevated body mass index (BMI) was found to be an independent predictor of lower in-hospital mortality, and it was studied in other diseases other than stroke in the literature [13-16]. Two recent studies have shown a relationship between anemia and thrombocytosis and stroke outcomes. Patients with ischemic stroke and anemia had increased LOS, while thrombocytosis in hemorrhagic stroke patients showed better outcomes in the literature [17,18]. There are no large studies except review articles evaluating the LOS in an acute inpatient setting for stroke patients in the US to the best of our knowledge, mainly about the type of discharge placement, health insurance, ethnicity, obesity, and hematological parameters. Several of these were related to social factors within the study population. This study aims to assess factors affecting LOS with a particular focus on the above social and hematological factors that were not analyzed well in the literature. 

## Materials and methods

In this retrospective study, we included adult patients (age >18 years) admitted to medical floors with acute stroke (admitted with first-ever stroke symptoms within 24 hours of presentation) during the study period from June 2021 to June 2023. We excluded pediatric and pregnant female patients in our study. This is a quality assessment, a single institute retrospective observational study. The study protocol was submitted to the Ethics Committee/Institutional Review Board (IRB) for review, and the waiver was received to proceed with the study before the beginning of the study. We measured the LOS for the study population and then divided the study population into two arms based on the LOS. In this study, the cut-off for dividing the study population was set as 10 days, as the literature for acute stroke LOS was variable (around 5-7 days). This is a quality assessment study on LOS, comparing results from literature/other institutes. Arm A included patients with LOS≤10 days, and arm B included patients with extended LOS>10 days. We compared the two arms in the study with the literature.

In this study, we collected various factors influencing length of stay (LOS), including age (categorized as under 65 years or 65 years and older), gender, ethnicity, and stroke type (ischemic or hemorrhagic). Patients with subarachnoid hemorrhage (SAH) and subdural hematoma (SDH) were excluded. Other variables included NIHSS scores, depression scores, type of medical floor admission, and medical comorbidities such as hypertension, diabetes mellitus, and obesity.

Additionally, procedural factors like neuro-interventions (e.g., thrombectomy), thrombolytic administration, and neurosurgical procedures were analyzed. Social determinants, type of placement, and independence with ambulation on admission (measured through activities of daily living (ADL) as per physical therapy assessments and medical records) were also considered. Further variables included health insurance status, medical complications, and hematological parameters such as hemoglobin levels and platelet counts. These factors were compared between two study groups and evaluated in the context of existing literature.

Statistical analysis was done using Graph Pad Prism 8 for all the study parameters after testing for normality using the Kolmogorov-Smirnov normality test. Arm A and Arm B populations were compared and analyzed using unpaired t-test, one-way Analysis of Variance (ANOVA), Chi-Square test for categorical and interval data, and Spearman correlation for two categorical parameters in the study as applicable. A two-tailed p-value of <0.05 was considered statistically significant in the study.

## Results

There were 483 patients with acute stroke treated at our institute from June 2021 to June 2023. Patients with subarachnoid hemorrhage (SAH) and subdural hematoma (SDH) were excluded from the study. The length of stay (LOS) was analyzed among the remaining 448 patients. Parameters with missing data on >10% of the study population (NIHSS score, depression score, type of medical floor admission) were excluded from the statistical analysis. The median LOS was 7.2±6.4 days for the study population, and the range was 0.1 to 49.7 days. Patient’s age at presentation, gender, type of stroke, procedures done, independence with ambulation before the stroke, type of placement, ethnicity, type of health insurance, body mass index (BMI)/obesity, and hematological parameters as hemoglobin level, platelet levels at presentation were analyzed (Table 1).

**Table 1:** Showing results of the study with all the parameters analyzed for acute stroke length of stay (LOS). Arm A included patients with LOS≤10 days and arm B included Patients with LOS>10 days. Anemia is defined as Hemoglobin (Hb)<12 gm%. Platelet counts are expressed as 1000/microliter. Results are expressed as absolute numbers or as median±standard deviation in the study.

Parameter	Arm A (N=357)	Arm B (N=91)	p-value
LOS in days	4.5±2.4	14.1±6.7	0.00001
Age in years	66±13.4	70±14.7	0.006
LOS in ≥65 years vs<65 years in the study 13.3±7.1 vs 6.9±5.4			0.005
Gender			
Male (number)	178	48	0.62
Female (number)	179	43	
LOS in males	4.5±4	15.1±4.5	<0.00001
LOS in females	4.7±2.6	18.8±9	<0.00001
LOS for study male vs female is 9.8 vs 11.7 days			<0.00001
Type of stroke			
Ischemic (percentage of study patients)	89% (N=318)	81% (N=74)	0.11
Hemorrhagic (percentage of study patients)	11% (N=40)	19% (N=17)	
LOS in ischemic stroke	4.9±2.3	16.5±6.4	<0.00001
LOS in hemorrhagic stroke	4.03±2.11	17±7.7	<0.00001
LOS for studying ischemic vs Hemorrhagic stroke is 9.3 vs 12.7 days			0.03
Procedure done (expressed as percentage of patients in the study)			
Yes	54% (N=192)	51% (N=46)	0.002
No	52% (N=186)	49% (N=45)	
LOS if procedure done	5.5±4.4	14.2±4.3	<0.00001
LOS if procedure not done	4.1±2.6	17.7±8.3	<0.00001
LOS for study population if procedure done vs. not done 7.2±4.2 vs 7.2±7.3 days			0.85
Independent with ambulation before admission (expressed as percentage of patients in the study)			
Yes	78% (N=278)	56% (N=51)	<0.00001
No	22% (N=78)	44% (N=40)	
Social factors			
Type of placement at discharge (expressed as percentage of patients in the study)			
Rehab hospital	19 % (N=68)	32% (N=29)	< 0.00001
Skilled nursing facility	5% (N=18)	19% (N=17)	
Hospice	1% (N=4)	8% (N=7)	
Death	5% (N=18)	1% (N=1)	
Home	70% (N=250)	27% (N=26)	
Type of insurance (expressed as percentage of patients in the study)			
Medicare/Medicaid	69% (N=246)	90% (N=82)	<0.0002
Other insurance	31% (N=11)	10% (N=9)	
Ethnicity (expressed as percentage of patients in the study)			
African American (AA)	49% (N=175)	55% (N=50)	0.36
White	50% (N=178)	42% (N=38)	
Other	1% (N=4)	3% (N=3)	
BMI	30±8.2	27.6±5.8	0.01
Obese (BMI >30) (expressed as percentage of patients in the study)	40% (N=143)	24% (N=22)	0.015
Hematological parameters			
Hemoglobin (Hb) level in gm% (mean±SD)	12.8±1.9	12.6±2.5	0.06
Anemia (expressed as percentage of patients in the study)	33% (N=118)	43% (N=39)	<0.00001
Normocytic anemia patients	81% (N=289)	64% (N=58)	0.004
Microcytic anemia patients	16% (N=57)	21% (N=19)	
Macrocytic anemia patients	3% (N=11)	15% (N=14)	
LOS in anemia patients (mean±SD)	3.3±3.7	17.2±6.8	<0.00001
Platelet count (expressed as 10^3^/microliter) (mean±SD)	258±70	231±94	0.58

**Figure 1:**
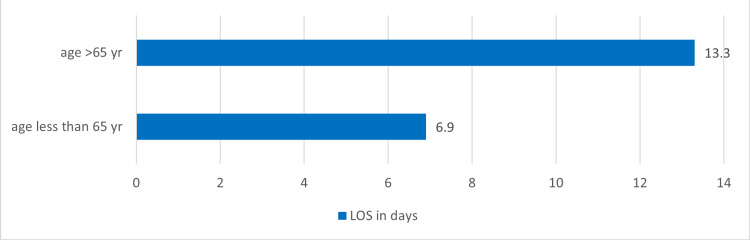
Showing median length of stay (LOS) in days in relation to the age of patients in the study.

Arm A had 357 patients with a median LOS of 4.5±2.4 days, ranging from 0.1 to 10 days, and arm B had patients with prolonged LOS >10 days, 91 patients with a median LOS 14.1±6.7 days, ranging from 10.1 to 49.7 days. The median age at presentation was 67 years, with a range of 22 to 101 years. There were 47% of patients aged ≤65 years. The LOS was higher in geriatric patients (aged >65 years) than younger patients, with a median LOS of 13.3±7.1 vs. 6.9±5.4 days, respectively, having a difference of about 6 days (p-value=0.005), as shown in Figure 1.

Arm A had a mean age of 66±13.4 years (range: 22-101 years), with 49% of patients being geriatric. The median length of stay (LOS) was slightly shorter among younger patients (4.6±2.5 days) compared to geriatric patients (4.7±2.3 days); however, this difference was not statistically significant (p=0.72).

In Arm B, the mean age was higher at 70±14.7 years (range: 24-99 years), with 67% of patients classified as geriatric. The median LOS was also lower among younger patients (16.4±7 days) compared to older patients (17.4±7.7 days), but this difference was not statistically significant (p=0.19).

**Figure 2:**
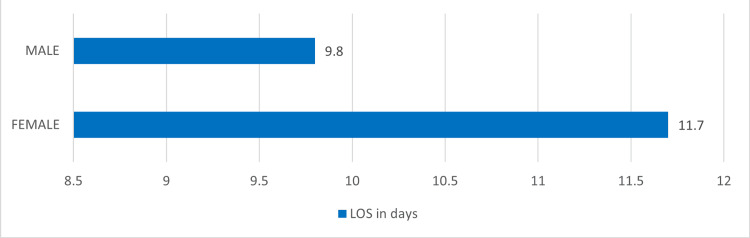
Showing median length of stay (LOS) in relation to the gender of the patient in the study.

Across the study, there were more male patients (54%) than females (46%). Notably, LOS was significantly longer in female patients (11.7 days) compared to male patients (9.8 days), with a statistically significant difference (p <0.0001), as depicted in Figure 2.

**Figure 3:**
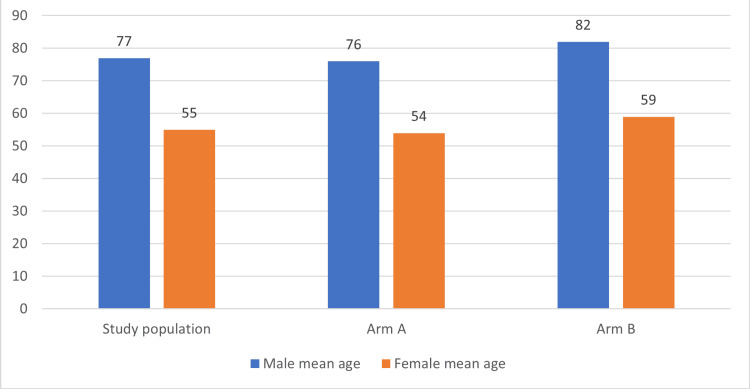
Showing age distribution in the study. The mean age of male and female patients in years, in the study and each arm has been shown here.

Arm A had 50% female patients with a median LOS of 4.7±2.6 days, while males in this arm had 4.5±4 days. Arm B had 53% female patients with a median LOS of 18.8±9 days, compared to 15.1±4.5 days for male patients. We analyzed the LOS in relation to age. Male patients were about 20 years older than female patients (p<0.0001) in both arms yet with lower median LOS, as shown in Figure 3.

**Figure 4:**
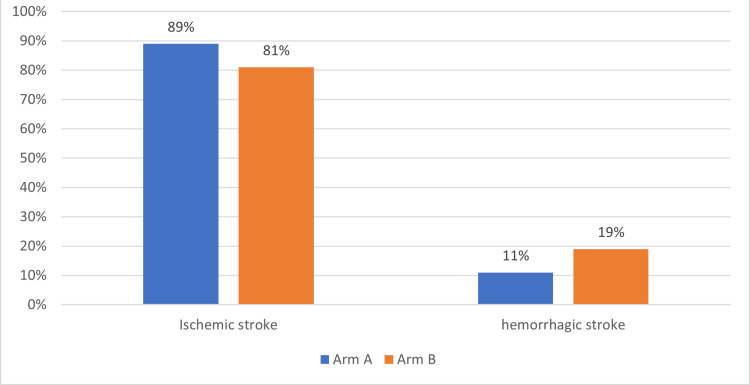
Showing the type of stroke in the study population (arm A and arm B) expressed in percentage.

This study analyzed the type of stroke/neurological diagnosis. These are summarized in Figure 4.

**Figure 5:**
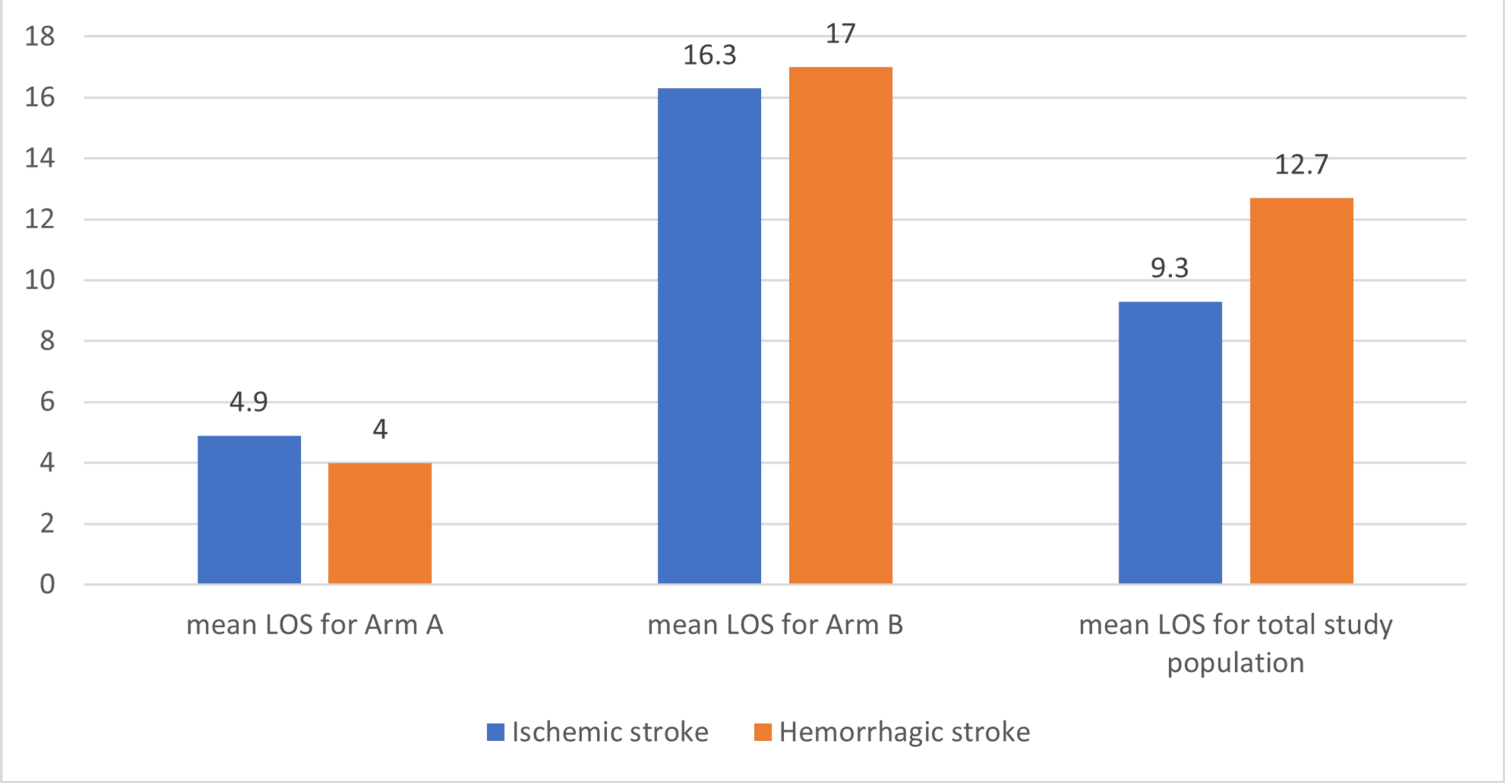
: Showing median length of stay (LOS) in days in the study population, in relation to type of stroke.

The LOS was longer in hemorrhagic vs. ischemic stroke in the study (p=0.03), as shown in Figure 5.

**Figure 6:**
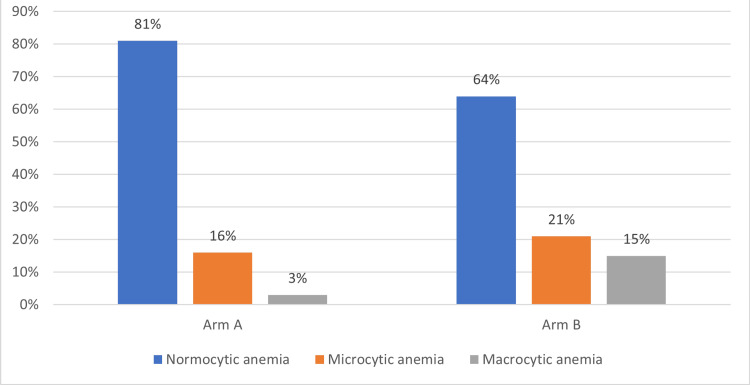
Showing the distribution of anemia at presentation in the study population, expressed as a percentage of patients.

There were 153 patients (about 35%) in the study who had procedure/s done. The LOS was the same or slightly longer in patients who had no procedure done (p=0.85). The LOS was analyzed in relation to the patient's functional status before the stroke. About 67% of the study population was independent of ambulation. The LOS was not significantly different among these groups (10.5 vs. 11.1 days), but there were more patients, 78% vs 56%, who were independent with ambulation before stroke admission in arm A than arm B (p<0.0001). Social factors additionally analyzed in the study, as shown in Table 1, showed that type of placement and insurance were statistically significant while ethnicity was not. We observed an obesity paradox with more obese patients in arm A but with lower LOS. We analyzed hematological parameters on admission. The mean hemoglobin level (Hb) level was not different between the arms, but the percentage of patients with anemia was 10% higher in arm B than in A (p<0.00001), as in Table 1. Most of the anemia patients had normocytic anemia, followed by microcytic anemia, as shown in Figure 6.

**Figure 7:**
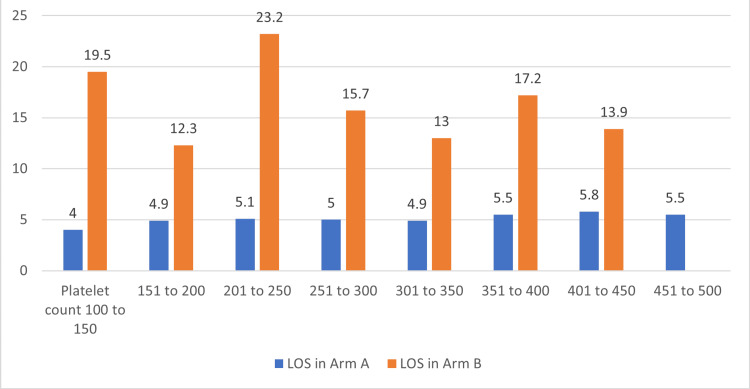
Showing median length of stay (LOS) with platelet counts in study arms A and B. Platelet count is expressed as 1000/microliter eg: 100 to 150 is 100 to 150 x 1000/microliter.

The platelet count at presentation was similar in both arms (p=0.58). We analyzed the LOS with a platelet range of 100000 to 500000/microliter in the study arms as most values (except for eight patients) were within this range at presentation. There was no significant correlation, with the Spearman correlation coefficient being 0.09 for arm A, while arm B had a negative correlation of 0.09 for platelet count with LOS in arm A (p=0.1) and arm B (p=0.3), as shown in Figure 7.

In Arm B, patients with prolonged length of stay (LOS) were further analyzed. Among them, 19% (n=17) had severe strokes with significant morbidity and either succumbed to the disease or were transitioned to comfort care/hospice. Of these, 7 patients died due to disease-related complications, while the remaining 10 were placed in comfort care or hospice. An additional 15% of patients (n=14) experienced delays in discharge due to social or placement issues, with an average delay of 10.8 days, ranging from 1 to 48 days. The remaining 66% of patients (n=60) had at least one additional medical complication, such as acute respiratory failure, infections (e.g., pneumonia or urinary tract infections), or the need for surgical procedures, including gastrostomy for feeding or craniotomy, all of which contributed to discharge delays.

## Discussion

This is a quality assessment study assessing the LOS in stroke patients at our institute. In our study, the median LOS was 7.2±6.4 days, and 80% of our study patients had LOS of less than 10 days, with a median of 4.6 days comparable to the literature (5-7 days) [2,3]. In our study, we had 10 days as cut off to divide the study population into two arms, as studies from literature on stroke patients showed a variable LOS with shorter LOS described as low as five days and few up to 12 or 15 days [4,7,9]. The LOS was not affected by age in other studies from the literature [5]. Also, the literature showed stroke patients aged >85 years had higher mortality even after discharge [11]. In our study, geriatric patients had higher LOS because our center is a stroke tertiary care center with a higher number of patients with comorbidities, which would delay the recovery. Also, geriatric patients would be prone to higher medical complications. The gender of the patient was not related to stroke LOS in some studies but was shown to be higher in males in others [5,6]. In our study, LOS was significantly higher in female patients. This needs to take into account the comorbidities and age of the patient to compare males and females, which was not done in other studies.

In our study when comparing males from females with age, the mean age was higher for males in both arms by about 20 years, but LOS was lower in males. Future studies should analyze if comorbidities with gender would affect LOS. In our study, most of our patients had thrombotic/embolic stroke comparable to literature (73% vs 80% respectively) [7]. In our study, hemorrhagic stroke patients had longer stays than ischemic stroke. Similar results were seen in the literature, and few studies in the literature showed no such difference [5,7]. Hemorrhagic stroke patients have worse outcomes than ischemic and are expected to have longer LOS. Patients with procedures (thrombolytics or mechanical thrombectomy) have lower mortality and improved outcomes, and our study showed similar results [19]. Patients who had procedures had similar or slightly shorter LOS in our study. This was not studied in the literature, but patients who had procedures probably had better performance than others, which could also explain the shorter LOS in them. The functional status before stroke affects LOS as per literature [4]. Patients independent with ADLs had faster recovery and fewer chances of infections like urinary infection, aspiration pneumonia, or deep vein thrombosis. In our study, the arm with short LOS had a higher number of patients who were independent with ADLs. Patients needing rehab placement had longer LOS, which is consistent with the literature [5]. In our study, 15% of long LOS patients were analyzed, and there was a delay of 1 to 48 days for discharging a medically stable patient due to social/placement issues to a rehab/skilled nursing facility (SNF).

Medical complications post-stroke increase the complication rate and mortality as per literature, which was consistent with the results of our study [7,8]. In our study, the majority (66%) of prolonged LOS patients had medical complications like infections during the same admission. Patients with Medicare/Medicaid insurance had longer LOS than other insurance types in our study. Similar results have been seen in trauma admissions but not evaluated on stroke patients before, to the best of our knowledge [10]. Ethnicity was analyzed, but most patients were African American in our study, with no significant difference affecting LOS in our study, unlike the literature [6,12]. Elevated BMI was found to be an independent predictor of LOS in patients admitted for stroke in our study. Obese patients had lower LOS. These results are similar to results seen in cardiovascular, chronic heart failure, and atrial fibrillation in literature [13-16].

Anemia prevalence and stroke outcomes were studied in a recent study done by Desai et al. [17]. Our study showed a similar percentage of stroke patients with anemia, and we have seen higher LOS for anemia patients. Platelet count had no impact on LOS in our study, unlike literature that shows better outcomes with thrombocytosis in hemorrhagic stroke [18]. Our study mostly involved ischemic stroke, and that could explain the results. Medical complications during hospitalization and severe stroke increase the LOS, as in the literature, and we found similar results in our study.

The merits of the study are that it’s a single institute study from a tertiary stroke center without much variability in treatment or patient population. Also, this is the first study evaluating the social and hematological factors in detail in acute stroke patients in relation to LOS. The demerits of the study are that we had incomplete data on NIHSS score, depression score, and type of medical floor admission, and these parameters were not analyzed. We also could not analyze the readmissions or survival data, which would be related to LOS.

## Conclusions

It was a quality assessment study, and our LOS for acute stroke was comparable to the literature. We did recognize that geriatric patients, female patients, patients with hemorrhagic stroke, not ambulatory before the acute stroke, social factors like discharge disposition to rehab or skilled nursing facility, Medicare/Medicaid insurance, hematological factor-anemia at presentation and medical complications during hospitalization, had a longer length of stay. Obese patients had lower LOS, and an obesity paradox was observed in acute stroke patients in the study. Future studies targeting these stroke patient subgroups would lower the LOS at our institute.
